# Event and Comment

**Published:** 1906-11-15

**Authors:** 


### DENTAL REGISTER.

Vol. LX. November 15, 1906. No. 11.

### EVENT AND COMMENT.

### Plea for an Improved Curriculum.

We give up largely of our space in this issue to the publication of a paper read before the Michigan Dental Association, by Dr. D. D. Smith, of Philadelphia. Our readers may think that we are getting over enthusiastic over the subject of oral prophylaxis, but we feel that this is one of the most important developments in the practice of our profession in recent years, and therefore worthy of all the space we have given it. We trust our readers may take the time to carefully consider this subject in the light of Dr. Smith's propaganda, as it is now, and will be for some time, one of the most important subjects up for discussion. The paper of Dr. Wright, and our comment on it, published last month, with this paper and its discussion, will set the matter fairly well before our readers and we would be pleased to open our pages to any further discussion that our readers may desire. The technique of the subject we shall hope to set forth from time to time in future issues.

